# Silver Nanoparticles Derived by *Artemisia* *arborescens* Reveal Anticancer and Apoptosis-Inducing Effects

**DOI:** 10.3390/ijms22168621

**Published:** 2021-08-11

**Authors:** Valentina Bordoni, Luca Sanna, Weidong Lyu, Elisabetta Avitabile, Stefano Zoroddu, Serenella Medici, David J. Kelvin, Luigi Bagella

**Affiliations:** 1 Department of Biomedical Sciences, University of Sassari, Viale San Pietro 43/b, 07100 Sassari, Italy; v.bordoni@studenti.uniss.it (V.B.); lusanna@uniss.it (L.S.); lvweidong1230@126.com (W.L.); avitabileelisabetta@gmail.com (E.A.); stefano.zoroddu@gmail.com (S.Z.); 2 Division of Immunology, International Institute of Infection and Immunity, Shantou University Medical College, Shantou 515011, China; dkelvin@jidc.org; 3 Department of Chemistry and Pharmacy, University of Sassari, Via Muroni 23, 07100 Sassari, Italy; sere@uniss.it; 4 Department of Microbiology and Immunology, Dalhousie University, 6299 South St, Halifax, NS B3H 4R2, Canada; 5 Centre for Biotechnology, Sbarro Institute for Cancer Research and Molecular Medicine, College of Science and Technology, Temple University, Philadelphia, PA 19122, USA

**Keywords:** cancer research, nanotechnology, silver nanoparticles, *Artemisia arborescens*, RNA-seq

## Abstract

The fight against cancer is one of the main challenges for medical research. Recently, nanotechnology has made significant progress, providing possibilities for developing innovative nanomaterials to overcome the common limitations of current therapies. In this context, silver nanoparticles (AgNPs) represent a promising nano-tool able to offer interesting applications for cancer research. Following this path, we combined the silver proprieties with *Artemisia* *arborescens* characteristics, producing novel nanoparticles called *Artemisia–AgNP*s. A “green” synthesis method was performed to produce *Artemisia–AgNP*s, using *Artemisia* *arborescens* extracts. This kind of photosynthesis is an eco-friendly, inexpensive, and fast approach. Moreover, the bioorganic molecules of plant extracts improved the biocompatibility and efficacy of *Artemisia–AgNP*s. The *Artemisia–AgNP*s were fully characterized and tested to compare their effects on various cancer cell lines, in particular HeLa and MCF-7. *Artemisia–AgNP*s treatment showed dose-dependent growth inhibition of cancer cells. Moreover, we evaluated their impact on the cell cycle, observing a G1 arrest mediated by *Artemisia–AgNP*s treatment. Using a clonogenic assay after treatment, we observed a complete lack of cell colonies, which demonstrated cell reproducibility death. To have a broader overview on gene expression impact, we performed RNA-sequencing, which demonstrated the potential of *Artemisia–AgNP*s as a suitable candidate tool in cancer research.

## 1. Introduction

Nowadays, cancer incidence is rapidly increasing, and it is considered to be the second most common cause of death worldwide [1]. Cancer is a group of diseases defined by a high proliferation index and alteration of several physiological cellular mechanisms. The main therapeutic treatments currently used are surgery, radiotherapy and chemotherapy. These treatments can lead to common complications, including severe side effects, incomplete tumor resection, and development of resistance [2,3]. Cancer nanomedicine is emerging as a new research field with the aim of offering potential nano-tools for oncological applications. The final goal of cancer nanomedicine is to provide early detection of tumors, accurate diagnoses, and personalized therapy [4,5]. The principal advantage of cancer nanomedicine consists of exploiting nano-sized particles that are able to act at a molecular level, on the road to more personalized medicine. In the last few years, several nanoparticles have been studied for cancer diagnosis and treatment, such as carbon nanotubes [6], paramagnetic nanoparticles [7], liposomes [8], gold nanoparticles [9], and many others [10]. Silver nanoparticles (AgNPs), thanks to their interesting physical-chemical properties, are becoming of great interest in cancer research. Silver is a noble metal with attractive biological properties, including anti-bacterial and anti-mycotic activities. Recently, some studies have shown that a large number of silver compounds have many effects on cancer cells [11]. Silver exhibits low toxicity but, at the same time, poor bioavailability due to the effective physiological mechanism of detoxification of the human body. Therefore, AgNPs represent an excellent solution to avoid this problem. Indeed, AgNPs can be internalized by cells through endocytosis and other up-take mechanisms, releasing Ag+ ions, which are the reactive species of silver, at the target sites [12]. Several studies have shown that AgNPs induce cytotoxicity through reactive oxygen species [13]. Nevertheless, to better understand the biological mechanism of AgNPs, further investigation is needed.

In the last few years, “green” synthesis is emerging as an alternative method to produce AgNPs. This approach is easy, inexpensive, and eco-friendly, since it is based on biosynthesis from plants, bacteria, and fungi extracts [14]. The “green” procedure involves the addition of a silver salt, commonly silver nitrate (AgNO_3_), to plant extracts so that the biomolecules present in the solution can reduce silver ions into metal nanoparticles. The main biomolecules implicated in this reaction are terpenoids, polyphenols, enzymes, and proteins. The shape and the dimensions of nanoparticles are influenced by the origin of the extracts, the temperature, and the pH of the solution. These characteristics affect the biological responses of AgNPs [15]. Moreover, biomolecules are present in the reaction medium, especially proteins and enzymes, which are then distributed on the surface of the particles as a layer called the “capping”. In addition, the capping influences the biological activity of AgNPs. Indeed, as suggested by several studies, nanoparticles presenting capping on their surface are more biocompatible compared to those that do not. The “capped” nanoparticles have shown less cytotoxicity but appear to be more efficient compared to “naked” nanoparticles [16,17].

Therefore, we used extracts of *Artemisia arborescens* to synthetize biologically active AgNPs. The employment of *Artemisia arborescens*, a typical Mediterranean plant, is based on the fact that it belongs to the genus *Artemisia*, whose species have been used worldwide as traditional or folk remedies to treat a wide number of conditions. For instance, one of the leading antimalarial drugs, artemisinin, was discovered in *A. annua* [18]. The same molecule and its synthetic derivatives are currently being tested against cancer with promising results [19,20,21]. In general, most *Artemisia* species show potential pharmacological effects due to the presence of useful terpenes and thujones in their leaves or flowers. Indeed, the pharmacological properties of *A. arborescens* are under investigation for several medical purposes [22] following its traditional consumption as a decoction to heal fever, cough, and malaria. Although many *Artemisia* species have been employed to prepare AgNPs for cancer treatment, to the best of our knowledge *A. arborescens* has not yet been investigated under this point of view. Thus, we tested their behavior towards cancer cells, and our findings are reported in the present paper. We have synthetized three different batches of *Artemisia–AgNP*s at different pH values (7, 8, and 9) and fully characterized them in order to understand their structural details and morphology. These nanoparticles have been tested on cancer cell lines, including HeLa and MCF-7. A dose-dependent cytotoxic action was found in cancer cells treated for 24 and 48 h with *Artemisia–AgNP*s. The major effects on cell viability were mediated by *Artemisia–AgNP*s synthetized at pH 7 compared to the others. After *Artemisia–AgNP*s treatment, we observed a G1 arrest and an increase in apoptotic cells. Through clonogenic assay, we found a high reduction in cell colonies after *Artemisia–AgNP*s treatment, demonstrating cell reproduction death. Furthermore, we performed RNA-sequencing to have a wide overview of the biological activity of *Artemisia–AgNP*s, with a regulation of gene expression and DNA damage response. Taken together, these preliminary data suggest the potentiality of *Artemisia–AgNP*s as a good anticancer candidate tool, which can be used to pave the way for further analysis in cancer research.

## 2. Results

### 2.1. Nanoparticles Characterization and XTT Analysis

Size is a fundamental factor in determining the biological activity of nanoparticles. In general, it has been observed that the smaller the size, the higher the activity [23]. Nevertheless, very small particles can exert toxic effects on cells: sometimes nanoparticles below 10 nm are not suitable for biological experiments because they can, for instance, cause hemolysis [24]. Nevertheless, the presence of capping on their surface can easily modulate their activity, making them less toxic and more active and biocompatible. Therefore, it is important to find a balance between activity and toxicity by tuning the dimensions of the nanoparticles. A good way to do this is to control the pH of the solution in which the nanoparticles are synthesized, knowing that in general higher pH corresponds to smaller particles [25]. Consequently, a procedure already in use was modified to control the dimension of the AgNPs by tuning the pH in solution [26]. Hence, three different batches of *Artemisia–AgNP*s have been prepared at distinct pH values (7, 8, and 9) in order to examine whether, as expected, the pH change was able to modify their size, but also to influence their capping and, therefore, their biological behavior. Ultracentrifugation at 13,000 rpm was introduced to increase the yield of the recovered AgNPs and to shorten the workout procedure, significantly improving the synthetic process.

**Figure 1:**
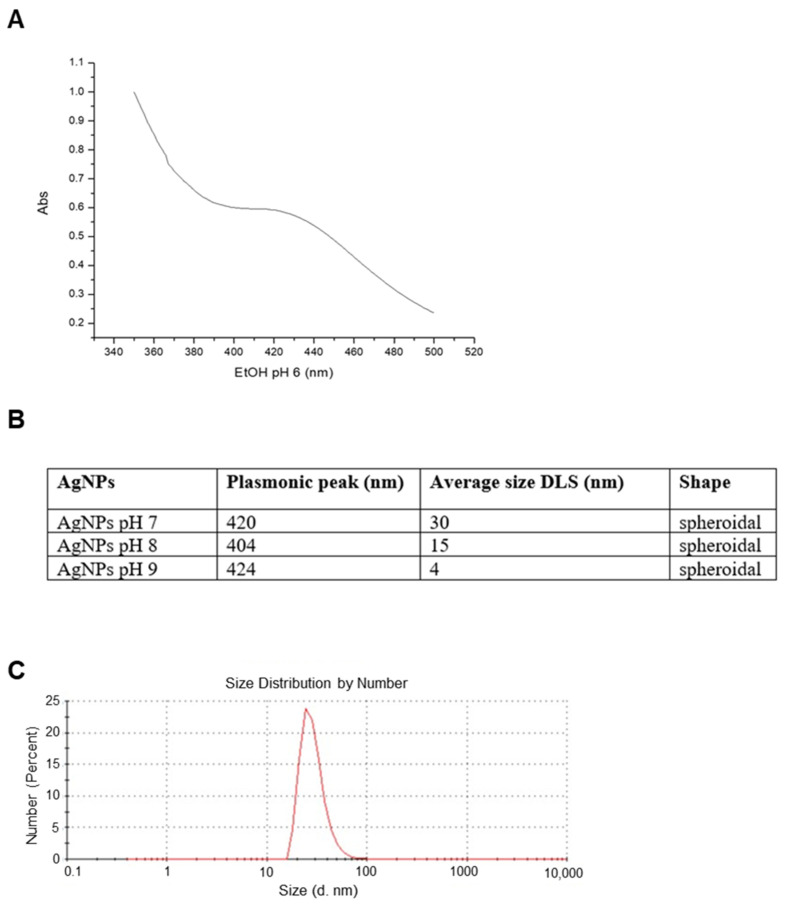
*Artemia*-AgNPs characterization. (**A**) Table indicating the major properties of all three nanoparticles. (**B**) Selected UV-vis spectrum for AgNPs at pH 7 showing the plasmonic peak at 420 nm. (**C**) Selected DLS measurements for AgNPs at pH 7.

A full characterization of the AgNPs was carried out in order to study their structural features. UV-vis spectra showed a plasmonic peak around 420 (Figure 1A), suggesting a spheroidal shape for these nanoparticles with a probable size between 10 and 30 nm [27]. DLS analysis showed that the size distribution of AgNPs changed according to the pH of the reaction solution. As expected, an increase in the pH determined a decrease in the hydrodynamic radius [28], which turned out to be around 30 nm for AgNPs at pH 7 (Figure 1B), around 15 nm for AgNPs at pH 8, and around 4 nm for AgNPs at pH 9. Measurements taken after weeks from their synthesis evidenced that the dimensions remained constant over time, indicating that these particles are rather stable.

**Figure 2:**
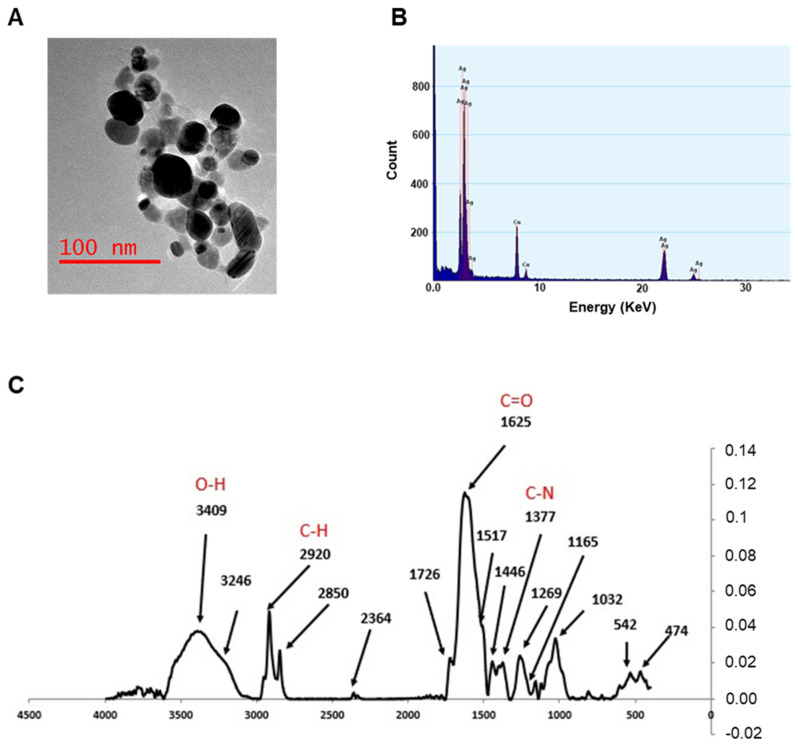
TEM analysis, EDX profile, and FTIR measurements. (**A**) TEM image for AgNPs at pH 7. (**B**) EDX profile showing the presence of elemental Ag (Cu signals are due to the grid used). (**C**) FTIR spectra of AgNPs synthesized at pH 7. Spectra were acquired in the range 400–4000 cm^−1^.

TEM analysis confirms the information on the size and morphology of the AgNPs, which were, in almost all cases, spheroidal in shape, with average dimensions well below 50 nm. The TEM image shown in Figure 2A displays the morphology of the nanoparticles obtained at pH 7, characterized by a spheroidal shape and no aggregation in water. EDX profile was used to evaluate the elemental structure of the nanoparticles (Figure 2B), underlining their silver composition. The other elements presented in the spectra are due to the holey carbon/copper grid used.

The FTIR measurements were performed by recording the signals in the range of 400–4000 cm^−1^ with a resolution of 4 cm^−1^, in order to acquire more information on the capping molecules. FTIR spectra from all samples were almost identical, indicating an analogous composition (Figure 2C). The bands observed suggest that amines, proteins, or (poly)phenolic compounds of *Artemisia* leaf extracts may be involved in the formation of the capping. A broad band from 3100 to 3600 cm^−1^ incorporates both OH and NH stretching frequencies, while the signals around 2900 cm^−1^ can be attributed to the sp3 CH stretching mode of hydrocarbon moieties, and those between 1630 (νC=O) and 1000 cm^−1^ are most probably due to amidic compounds (i.e., proteins and enzymes) or amines.

The characterization of the three batches of AgNPs showed that, while they were all spheroidal in shape and shared the same capping molecules, the pH at which they were obtained was actually able to influence their dimensions, with a higher pH corresponding with smaller nanoparticles as expected.

**Figure 3:**
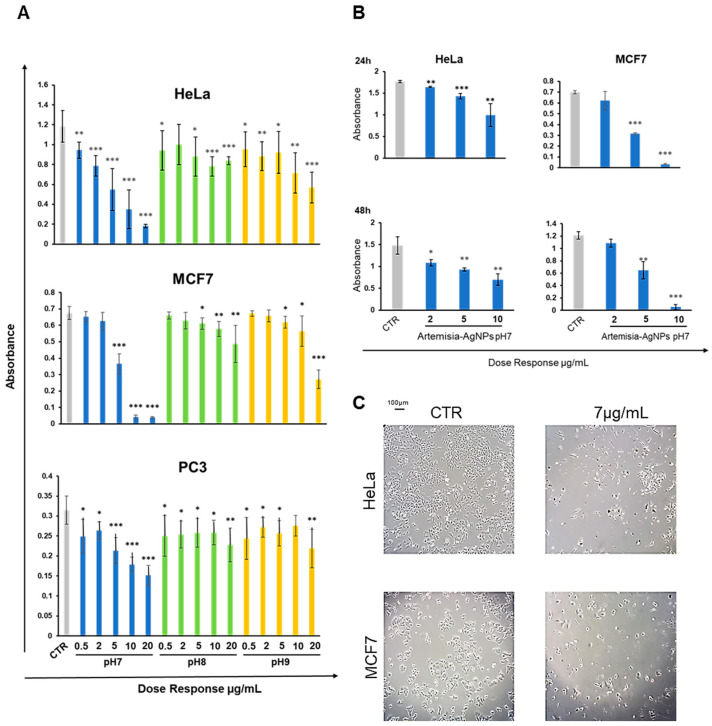
XTT assay. (**A**) HeLa, MCF-7, and PC3 were treated with increasing doses (0.5–20 µg/mL) of each Artemisia–AgNPs (pH 7, pH 8, and pH 9). An XTT assay was performed to evaluate cell viability. (**B**) XTT assay of Artemisia–AgNPs pH7 on HeLa and MCF-7. HeLa and MCF-7 were cultured with 2, 5, and 10 µg/mL of Artemisia–AgNPs pH 7. Cell viability was evaluated using XTT assay. (**C**) Microscope images. HeLa and MCF-7 treated for 24 h with 7 µg/mL of Artemisia–AgNPs pH 7. Data were analyzed using Student’s *t* test, * = *p* value < 0.05, ** = *p* value < 0.01, *** = *p* value < 0.001.

To check the antitumor effects of our AgNPs, we treated different cell cancer lines (HeLa, MCF-7 and PC3) with increasing doses (from 0.5 to 20 µg/mL) of Artemisia–AgNPs (pH 7, pH 8 and pH 9) in order to evaluate cell viability by XTT assay. We observed a dose-dependent anti-proliferative effect of Artemisia–AgNPs. The decrease in viability was more relevant for the Artemisia–AgNPs pH7 compared to the others, with a decrease in cell viability in a dose dependent manner (Figure 3A). Other studies demonstrated the ability of AgNPs to inhibit cell proliferation, inducing cell death [29,30]. Moreover, it was shown that the cytotoxic action is influenced by size and surface area of AgNPs [31]. Regarding the distinct action of AgNPs due to the different sizes, recent evidence suggests that the appropriate size for cellular uptake, in order to achieve the greatest biological activity of nanoparticles without the risk of hemolysis, is around 40–50 nm [32].

Moreover, as reported in Figure 3A, the anti-proliferative activity of Artemisia–AgNPs pH7 is more evident on HeLa and MCF-7 compared to PC3 (Figure 3A). In accordance with our results, Sadegh et al. observed that AgNPs synthesized using green synthesis showed more selective activity against an MCF-7 cell line [33].

Based on these data, we selected Artemisia–AgNPs pH7 for further analysis in order to clarify their specific effects on HeLa and MCF-7. Therefore, to validate the cytotoxic effects, XTT assay was repeated at 24 h and 48 h, using concentrations of 2, 5, and 10 µg/mL of Artemisia–AgNPs pH 7. As showed in Figure 3B, Artemisia–AgNPs pH7 revealed a high anti-proliferative effect, especially at the concentration between 5 and 10 µg/mL. Thereby, for the following experiments, we considered 7 µg/mL of Artemisia–AgNPs pH7 to be the ideal concentration to significantly reduce cell viability. The microscope images in Figure 3C clearly show the cells after 24 h of treatment with 7 µg/mL of Artemisia–AgNPs pH7. Compared to the control, most cells suffered when detached from the plate and died after treatment.

### 2.2. Cell Cycle Impact

**Figure 4:**
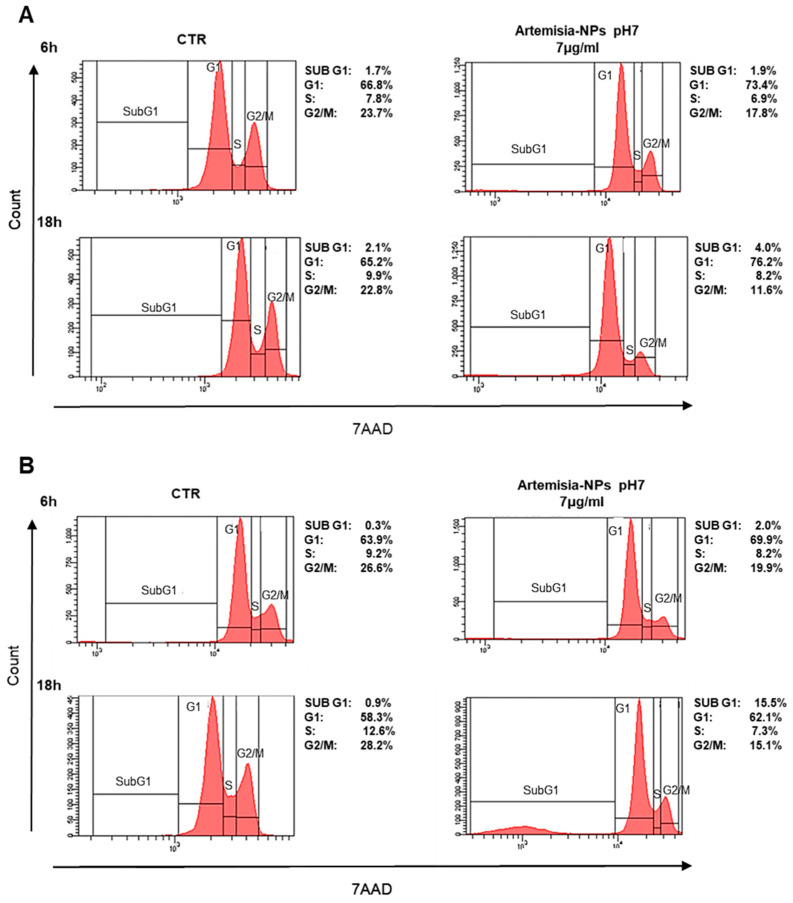
Cell cycle analysis. (**A**) HeLa cells were treated with 7 µg/mL of Artemisia–AgNPs pH 7 or left untreated. The cell cycle was evaluated after 6 h and 18 h by flow cytometry. (**B**) MCF-7 cells were treated to the same condition as HeLa and cell cycle effects were evaluated by flow cytometry.

In order to evaluate the impact on the cell cycle, cells were treated with 7 µg/mL of Artemisia–AgNPs for 6 h and 18 h. Interestingly, a significant reduction in the number of HeLa cells in G2/M phase was found after only 6 h of treatment in comparison to control samples (Figure 4A). At 18 h of incubation, the arrest of cells in the G0/G1 phase was evident (Figure 4A). Similar results were found in MCF-7 cell lines treated with Artemisia–AgNPs (Figure 4B). Especially, after 18 h of incubation, the percentage of cells in the G2/M phase strongly decreased in association with an increase in G0/G1 phase. Moreover, we observed a relevant increment of the SubG1 phase, an index of cellular death (Figure 4B). The impact on cell cycle of cancer cells mediated by AgNPs was analyzed by other studies. The exposition to 10–50 μg/mL of AgNPs for 24 h determined a relevant increase in the SubG1 population on HeLa cells and cells were not able to go through G2 checkpoint [34]. In contrast to our results, other authors studied AgNPs on lung epithelial cells and glioblastoma cells, observing an increase in G2/M-phase cells accompanied by a decrease in the G1 population after treatment [35]. This divergence in cell cycle data may be due to different factors including cell lines, distinct synthesis of AgNPs, and presence of capping able to change the biological behaviors of AgNPs. Nonetheless, further investigations are needed to better understand the cell cycle effects mediated by AgNPs.

### 2.3. Artemisia–AgNPs Induce Apoptosis and Inhibit Colony Formation in Cancer Cells

**Figure 5:**
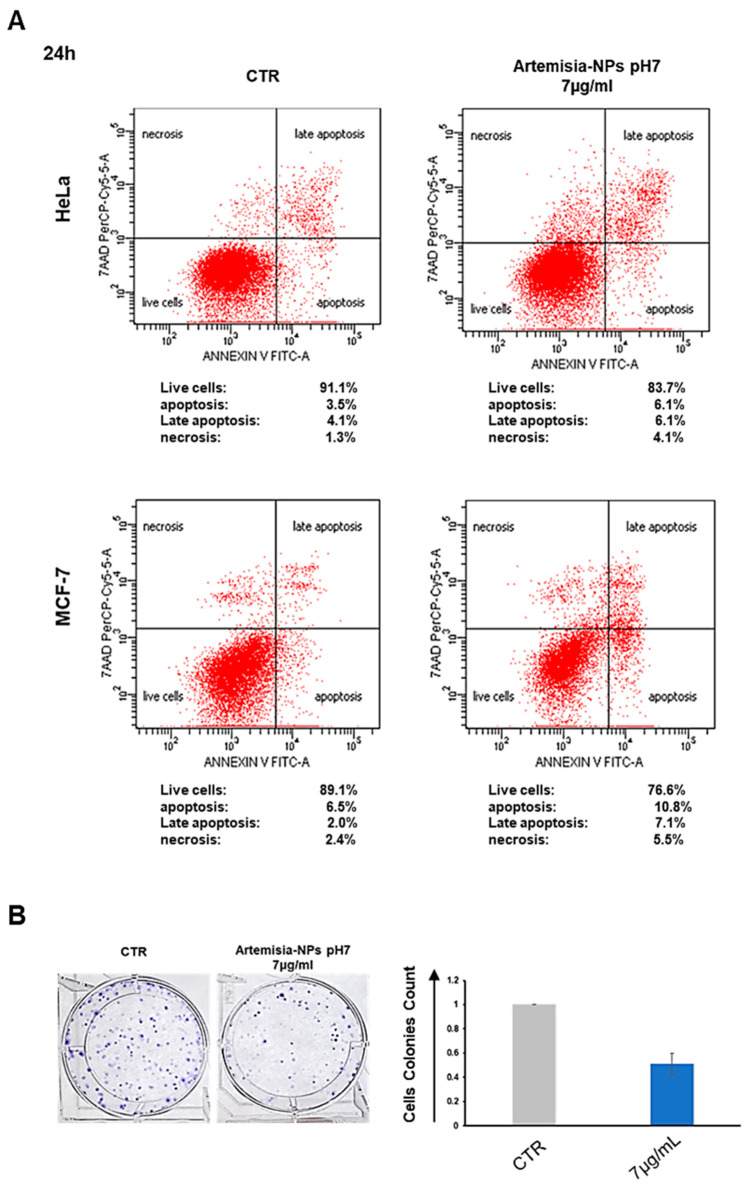
Apoptosis and cell colony formation assays. (**A**) Apoptosis/Necrosis assay. On the left control samples, while on the right samples treated with 7 µg/mL of Artemisia–AgNPs pH 7. Above HeLa cells and below MCF-7 cells. Cells were stained with Annexin V/7aad and analyzed by flow cytometry. (**B**) Clonogenic assay. After the formation of the first colonies, cells were treated with 7 µg/mL of Artemisia–AgNPs pH 7. Cell colonies were stained and counted.

Following cell cycle analysis, in order to discriminate between apoptotic and necrotic cells, an Annexin V/7aad assay was performed. To enhance the dead cell number, cells were incubated for 24 h with 7 µg/mL of Artemisia–AgNPs pH7. We determined that Artemisia–AgNPs induced significant apoptotic effects in both cell lines, with a slight increment of necrotic cells (Figure 5A). In line with our data, various studies have demonstrated the ability of AgNPs to induce apoptosis in several cancer cell lines [36,37]. In any case, treatment with high doses of AgNPs may lead to a prevalent necrotic effect [38].

Moreover, we employed a clonogenic assay to assess the ability of Artemisia–AgNPs pH7 to arrest cell colony formation. Figure 5B clearly shows the clonal growth inhibition mediated by 7 µg/mL of Artemisia–AgNPs pH7.

### 2.4. Artemisia–AgNPs Impact on Gene Expression

**Figure 6:**
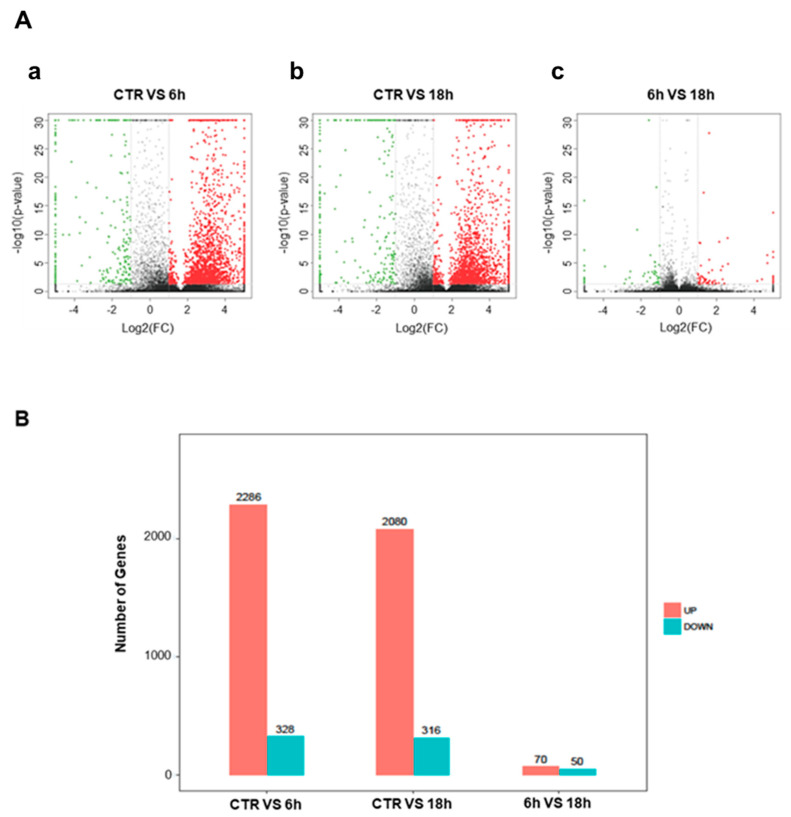
Differentially expressed genes. (**A**) Volcanic map of differential genes. The DEGs set after 6 h and 18 h of Artemisia–AgNPs treatment. Red dots indicate up-regulated genes, green dots represent down-regulated genes, and black dots indicate genes with no significant difference. (|logFC| ≥ 2 and *p* value < 0.05). a: CTR vs. 6 h; b: CTR vs. 18 h; c: 6 h vs. 18 h. (**B**) Histogram of differential genes. The DEGs set disturbed by Artemisia–AgNPs treatment (|logFC| ≥ 2 and *p* value < 0.05).

To elucidate the mechanism of action of Artemisia–AgNPs, we performed RNA-sequencing on HeLa cells in order to have a wide overview of the impact on gene expression. After 6 h and 18 h of treatment, we observed several differentially expressed genes (DEGs), using parameter the log2 fold-change values and *p*-values as reference indicators for significant difference (Figure 6A). Figure 6B shows in detail the gene variations: 2286 up-regulated and 328 down-regulated genes after 6 h of treatment, while after 18 h of treatment 2080 up-regulated and 316 down-regulated genes are shown.

**Figure 7:**
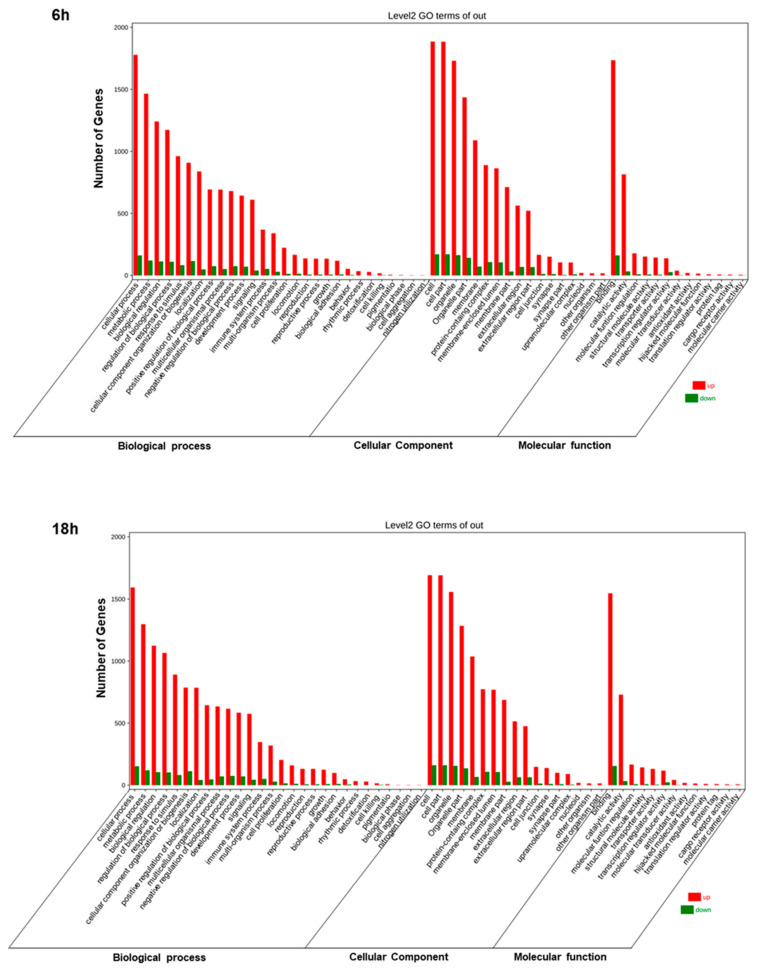
GO analysis. GO annotation for 6 h and 18 h of treatments of DEGs. The number of enriched genes is presented.

Afterward, we employed OmicShare tools, an online software, for the gene ontology (GO) analysis, enabling the study of the functions of the differentially expressed genes (DEGs). In detail, through these tools it is possible to evaluate the cellular components, biological processes, and molecular functions correlated with DEGs. We found similar results at 6 h and 18 h. Artemisia–AgNPs appear to work at an intracellular level, with enriched genes mainly expressed in organelles and their membranes (Figure 7). Regarding the biological processes, the main up-regulated genes were correlated with metabolic processes and biological regulation. The molecular functions of DEGs were mainly involved in binding, catalytic activity, and molecular function regulator.

**Figure 8:**
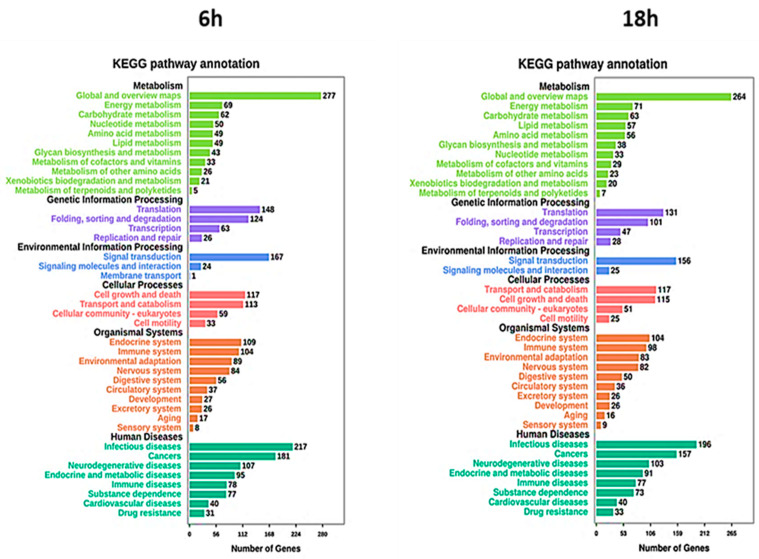
KEGG analysis. The pathways involved in the DEGs were analyzed using KEGG analysis. The number of enriched genes and pathway terms are presented.

To further characterize the differentially expressed genes, investigating the metabolic and signal transduction pathways involved, KEGG pathway analysis was performed. The analysis showed a strong association between DEGs and cellular metabolism, translation, and folding processes (Figure 8). Moreover, several DEGs were also correlated with signal transduction and cell growth and death. Regarding human diseases, the DEGs correlated with Artemisia–AgNPs treatment are mainly enriched in infections and cancers.

**Figure 9:**
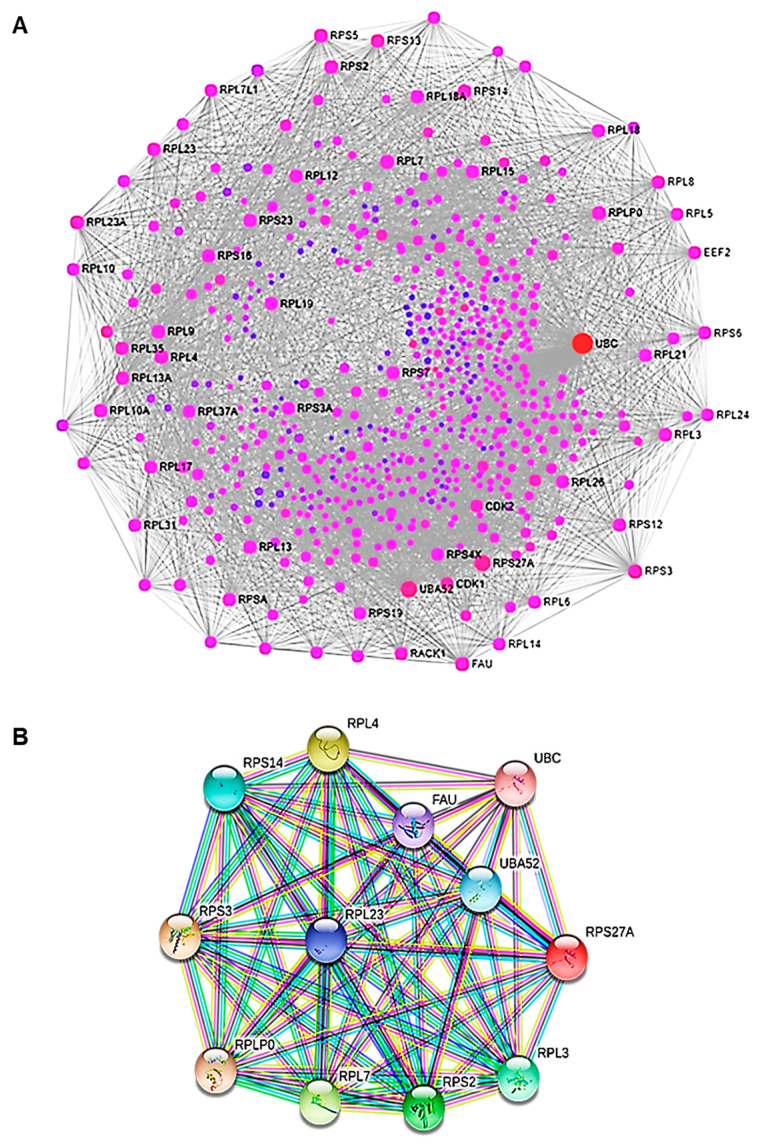
PPI analysis. (**A**) The PPI network of DEGs. The nodes represent the differential genes and the connections between the nodes reveal the interactions between the genes. The size of the nodes indicates the degree of the genes, and the different colors represent the distinct gene expression (red are up-regulated genes and violet are less up-regulated). (**B**) The PPI network of hub genes. Co-expression network with top hub DEGs.

To investigate the protein–protein interaction (PPI) network, we used an online mapping tool (NetworkAnalyst3.0) (Figure 9A). The PPI network analysis led to the evaluation of the top 15 genes, classified as hub genes by degree. The following are the names of the hub genes: UBC, UBA52 (over-expressed during hepatoma cell apoptosis), RPS27A (cell malignant transformation), RPS3 (cell apoptosis regulation), FAU (down-regulated in human prostate, breast, and ovarian cancers), RPL7 (cell apoptosis regulation), RPL23A, RPL4 (self-translation regulation—*E. coli*), RPLP0 (tumor progression, invasion, metastasis), RPS5, RPL9, RPS14, RPS2, RPL3, and RPL23. These genes encode for ubiquitin-ribosomal proteins, which are involved in a variety of biological events, including cellular mechanisms correlated with cancer formation and progression. Ubiquitin is a highly conserved regulatory protein that covalently binds proteins, leading to post-translational modification of proteins. Ubiquitination is a crucial event for cell cycle progression and cell proliferation. Alterations of this process are associated with cancer development [39]. In order to clarify the association between the hub genes, we also performed a PPI analysis of the hub genes (Figure 9B). Therefore, through the functional and pathway enrichment analysis, we identified the involvement of these 15 hub genes in several biological processes, including regulation of gene expression, DNA damage response, genome nucleotide-excision repair, and in ribosomes (Table 1).

## 3. Discussion

Although research has significantly advanced, the fight against cancer remains one of the main challenges for the scientific community. The current treatments of cancer have shown several problems, including side effects, poor selectivity, and drug resistance. To overcome these limitations, nanotechnology is emerging in this field in order to develop innovative tools with the aim of establishing a more effective and selective action against cancer cells [40]. In the last few years, a wide variety of nanomaterials and nanoparticles have been investigated for their potential use in oncology [41,42,43]. Nanotechnology can offer the opportunity to fight cancer with several approaches, including drug delivery, imaging, diagnosis, and therapy. For instance, nanoparticles can be useful to improve pharmacokinetic and pharmacodynamic profiles of existing anticancer compounds and combining cancer therapies [44,45]. In this context, several metallic nanoparticles have shown considerable interesting antitumoral activity against human cancer cells [46]. Among them, AgNPs, thanks to their peculiar physical-chemical features, have shown intrinsic antiproliferative activity [47]. AgNPs were investigated to develop a new-generation diagnostic and treatment tool for cancer [48]. Through a “green” synthesis approach, it is possible to produce stable AgNPs in an easy and inexpensive way.

The physicochemical characteristics of AgNPs, including shape, size, and coating, influence the biological behaviors [49]. Nanoparticles with sizes less than 100 nm exhibit a higher cytotoxic capacity and higher capacity to escape from a mononuclear phagocytic system. To evaluate the biological activity, we produced three different types of *Artemisia–AgNP*s. *Artemisia–AgNP*s synthesized at pH 7 showed higher activity, with strong cell growth inhibition and G1 cell cycle arrest accompanied by apoptotic induction in cancer cell lines. Other recent studies investigated the anti-proliferative effects of AgNPs synthesized using Artemisia genus plants and found cytotoxic activity on cancer cells in a dose–response manner [50,51]. In accordance with our results, Fard et al. found apoptotic action mediated by AgNPs synthesized using *Artemisia* increased the expression of apoptotic genes such as Bax and Bcl2 [52].

In order to deeply investigate the effects of *Artemisia–AgNP*s, we performed RNA-seq. The transcriptomic analysis showed several up- and down-regulated genes after *Artemisia–AgNP*s treatment. The main DEGs were related to organelle parts and their membranes, linked to metabolic processes and biological regulation. AgNPs induced cytotoxicity through various cellular processes. The uptake of AgNPs influences their cytotoxicity, leading to the entry of AgNPs into the cytosol, mitochondria, and nucleus. Once inside the cells, AgNPs can lead to cell death through several mechanisms, such as by damaging the mitochondria, decreasing ATP content, increasing reactive oxygen species (ROS) production, and damaging DNA [53]. AgNPs can induce cellular apoptosis by activating p53, *p*-Erk1/2, and caspase signaling [54]. Moreover, AgNPs revealed a more selective cytotoxic effect against cells of a breast cancer subtype compared to non-tumorigenic cells derived from breast, liver, kidney, and monocyte lineages [55].

In this study, we combined the functional assays and RNA-Seq, observing that *Artemisia–AgNP*s induced cell apoptosis and inhibited cell proliferation through cell-cycle arrest, regulation of gene expression, and DNA damage response. The KEGG analysis revealed that DEGs in *Artemisia–AgNP*s treatment are mainly enriched in infections and cancers. The hub genes raised from PPI analysis were genes coding for ribosomal proteins and ubiquitin family proteins. UBA52 and RPS27A genes code for a single copy of ubiquitin fused with ribosomal proteins 60S ribosomal protein L40 and ubiquitin-40S ribosomal protein S27A, respectively [56]. Indeed, L40 and S27A are ribosomal proteins synthesized as a ubiquitin C-terminal extension protein [57]. L40 is involved in regulation of gene expression and stress response. Meanwhile, 27A has a role in ribosome biogenesis and post-translational modifications of proteins, as well as extra-ribosomal functions. UBA52 and RPS27A genes were found to be differently over-expressed during hepatoma cell apoptosis [58]. Furthermore, in several studies, RPS27A was found to be correlated with cell malignant transformation and enhanced chemoresistance [59,60]. Additionally, hub genes include RPS3, which is a ribosomal protein involved in DNA repair endonuclease. This protein plays a role in cell apoptosis regulation. RPS3 induces apoptosis, whose signal is executed through the activation of caspase-8 followed by caspase-3 activation [61]. RPL7 is also involved in cell apoptosis regulation. Moreover, RPLP0 was found to be involved in tumorigenesis, cellular transformation, invasion, and metastasis. RPLP0 encodes for a ribosomal protein that can interact with P1 and P2 to form a pentameric complex consisting of P1 and P2 dimers and a P0 monomer. Different studies have shown the over-expression of RPLP0 in tumor tissue, especially gynecological tumors, breast cancer, and gastric cancer [62,63]. Specifically, several of the down-regulated genes analyzed by RNA-seq play important roles in cell proliferation, migration, and tumor progression. A recent study showed that down-regulation of RPS27A inhibits cell growth and induces apoptosis in Caco-2 cells [64]. Furthermore, another study shows that knockdown of RPS3 inhibits proliferation, survival, migration, invasion, and increases apoptosis in Caco-2 cells. This finding correlates with a decrease in p53 protein levels in RPS3-knockdown cell lines. Interestingly, knockdown of p53 in Caco-2 cells did not affect RPS3 levels, indicating that p53 may be a downstream target of RPS3 [65]. A recent study highlights that down-regulation of RPLP0 decreases cell viability and cell proliferation and increases cell death in HtTA cervical cancer cells. Particularly, down-regulation of RPLP0 levels correlates with decreased expression levels of cell cycle-associated and anti-apoptotic proteins: Cyclin D1, Cyclin E1, Cdk4, and BCL-2, along with increased expression levels of pro-apoptotic BAX [66]. Lastly, the PPI analysis identified the main hub genes as genes implicated in the control of multiple cellular processes correlated with cancer development, including cell cycle control, apoptosis regulation, control mechanisms of transcription and translation, and DNA repair.

In closing, we demonstrated the potential anti-proliferative and anti-cancer activity of *Artemisia–AgNP*s. Bio-organic active agents of *Artemisia arborescens* and Ag+ ions may work in a synergic way to enhance the anticancer properties of AgNPs. These outcomes provide new insights into molecular mechanisms and open doors for future investigations aimed at developing innovative nanoscale platforms, which will help us to achieve the longed for medical revolution of nanomedicine.

## 4. Materials and Methods

### 4.1. Artemisa-AgNPs Synthesis and Characterization

*Artemisia arborescens* was extracted in hydroalcoholic solutions (50:50) by heating 10 g of fresh leaves in the solvent mixture at 50 °C for 30 min under gentle stirring. 100 mL of the plant extracts were then used to reduce silver nitrate (AgNO_3_, 340 mg, 2 mmol) in 900 mL of milliq water at 30 °C. *Artemisia–AgNP*s had to be synthetized at three different pH values (7, 8, and 9), thus the pH was changed accordingly, by adding a few droplets of NH_4_OH concentrate solutions. The color of the mixture changed from light green to brown, indicating the formation of AgNPs. The color intensity changed with the pH. After 24 h, the reaction was completed and the AgNPs were then recovered by ultracentrifugation (13.000 rpm), dried under air, and characterized.

UV-vis spectra have been recorded on a T80+ UV-visible spectrophotometer (PG Instruments Ltd., Leicester, UK), in a quartz cuvette with a cell length of 1 cm in the frequency range 600–350 nm. The concentration of the solution was 1 mg/mL.

FTIR spectra have been recorded on a FTIR VERTEX 70 spectrophotometer (Bruker, MA, USA) in the frequency range 4000–400 cm^−1^ with a resolution of 4 cm^−1^ on KBr pellets (1:100 AgNPs).

The determination of the average size distribution of the nanoparticles was performed using the spectroscatterer Zeta Sizer Nano-S90 (Malvern Panalytical, Malvern, UK). To prepare the sample, the dried AgNPs powder (1 mg) was dispersed in 10 mL of distilled water.

Transmission electron microscopy analysis (TEM) was used to study the size and morphology of *Artemisia–AgNP*s. The images were obtained using the instrument FEI TECNAI G2 F20 TWIN in combination with an accelerating voltage of 200 Kv. In order to investigate the elemental structure of the AgNPs, energy dispersive X-ray spectroscopy was performed. AgNPs samples were dispersed in ethanol and sonicated for 30 min. One drop of nanoparticle suspension was deposited on a holey carbon/copper grid and measured.

### 4.2. Cell Cultures

HeLa (cervix adenocarcinoma), MCF-7 (breast cancer), and PC3 (prostate cancer) were purchased from ATCC. Passage of cells was between 7–10 [67,68]. Cells were cultured in Dulbecco’s modified Eagle’s medium (Gibco) containing 1% penicillin/streptomycin solution and 10% fetal bovine solution (FBS) (Gibco). All cell lines were grown at 37 °C in 5% CO_2_ in a humidified incubator.

### 4.3. Proliferation Assay (XTT)

The XTT assay (Cell Proliferation Kit II, Roche, Basel, Switzerland) was used to evaluate cell viability. Cells were seeded in 96-well plates at a density between 1500 and 2000 cells/well depending on the different size, phenotype, and cellular population doubling. After 24 h from seeding, cells were treated with increasing concentrations (from 0.5 to 20 µg/mL) of the three different *Artemisia–AgNP*s. Untreated cells were used as control. An XTT assay was performed following 24 and 48 h of treatment. In the presence of an electron-coupling reagent, the tetrazolium XTT salt was converted to formazan. Considering 100 µL/well as the final volume, the mix was prepared as follows: 0.5 µL of XTT electron coupling reagent, 25 µL of labeling reagent, and 74.5 µL of medium. The mix was used to incubate cells for four hours at 37 °C. After incubation, the absorbance was measured at 490 nm using a spectrophotometric plate reader. All experiments were carried out in triplicate.

### 4.4. Cell Cycle Analysis

To evaluate cell cycle impact, HeLa and MCF-7 were seeded in 6-well plates (5 × 105 cell/well). After 24 h from seeding, cells were treated with 7 µg/mL of *Artemisia–AgNP*s pH 7 or left untreated. The cells were incubated for 6 and 18 h and after that, supernatants and adherent cells were collected before centrifuging at 500 *g* for five minutes. Thus, the pellets were washed with PBS and centrifuged again at the same conditions as before. To fix the cells, we resuspended the pellets in 200 μL of PBS, then we used 70% ice-cold ethanol in agitation on a vortex. The samples were kept at −20 °C for a maximum of seven days. Before the analysis, fixed cells were washed twice with PBS, resuspended in 200 μL of PBS, and incubated with 20 μL of 7aad label for 20 min under dark conditions. The cell cycle was evaluated using FACS CANTO II (BD Biosciences, Milan, Italy) by collecting 20,000 events. The data were analyzed using DIVA software (BD Biosciences, Milan, Italy).

### 4.5. Necrosis and Apoptosis Evaluation

AnnexinV/7aad labeling was used to discriminate live cells, apoptotic cells, and necrotic cells. After 24 h of incubation with 7 µg/mL of *Artemisia–AgNP*s, the supernatants and adherent cells were collected. After centrifuge, the pellets were resuspended in 50 μL of Annexin V 1× buffer and stained with Annexin V/7aad. Following 20 min of incubation in the dark, 200 μL of Annexin V 1× buffer was added in each sample and the cell fluorescence was measured by flow cytometry (FACS CANTO II BD Biosciences, Milan Italy), collecting 20,000 events. DIVA software was used to analyze the data.

### 4.6. Colony Assay

HeLa cells were seeded in 6-well plates at a concentration of 100–200 cells/well. Cells were treated after formation of the earliest colonies, usually following five–seven days from seeding. Cells were incubated at 37 °C and maintained in culture for about two weeks, changing medium with or without AgNPs every three days, until control samples had formed enough visible colonies. To visualize cell colonies, the cells were stained with 2 mL of 6.0% glutaraldehyde and 0.5% crystal violet for 30 min. After washing with ddH20, the stained colonies were counted, and treated samples were compared with controls.

### 4.7. mRNA Extraction and Preparation of mRNA-Seq Library

HeLa cells were seeded in 6-well plates at a density of 2 × 105. After 6 h and 18 h of treatment with 7 µg/mL of *Artemisia–AgNP*s, cells were collected by centrifugation at 500 g and the pellets were washed twice with PBS. Total RNA was extracted using RNeasy MINI Kit (Qiagen, Hilden, Germany) following manufacturer’s protocol and the RNA quality was assessed by Agilent 2100 Bioanalyzer. Using RNaseH digestion and ribosomal-depleted RNA, we removed the rRNA from total RNA, which was purified by VAHTSTM RNA Clean Beads.

cDNA was synthetized using random primers and, to label the second strand, a dUTP was incorporated. The fragments were purified using magnetic bead, obtaining fragments of 150–200 bp. The libraries were sequenced on the Hiseq-PE150.

### 4.8. Quality Control and Gene Analysis

First, we checked the raw sequencing data obtained in terms of quality with fastQC. Trimmomatic software was used to filter the low-quality and unmeasured bases in order to obtain final clean data. To provide the reference of genome and gene annotation files, we downloaded the hg38 reference genome (grch38.p12. genome) and gene annotation GTF (GRCh38, version 30, Ensembl 96) in the gencode database. RSEM software was used to quantify the transcript abundances from RNA-Seq data. The reference genome and gene annotation files were used for digital gene expression analysis. After that, we obtained expression level analysis of clean reads by RSEM and we used STAR alignment in order to achieve gene expression results from samples. The number of readings segments that mapped to each gene sequence was calculated by FPKM (fragments per kilobases per million reads), considering the gene length and the sequencing depth. FPKM is a normalized estimator that allows us to estimate the expression value of a gene. We performed DEseq to analyze the difference in gene expression between the control group and treated samples. The differential gene screening criteria were based on the parameter values log2 fold-change and *p*-value (|logFC| ≥ 2 and *p*-value < 0.05).

### 4.9. GO Enrichment and KEGG Pathway Analysis

Since the functional and pathway enrichment analysis of differentially expressed genes (DEGs) was needed in order to clarify the biological processes and molecular mechanisms, GO enrichment and KEGG pathway analysis were applied for the identification of key genes and pathways involved. GO stands for Gene Ontology database, which is an international classification system standardized for gene functions. The classification of genes is organized into ontologies (or graph structures), which are grouped into three categories: molecular function, cellular component, and biological process. Gene Ontology database is usually used for annotating and sequencing gene products. Therefore, GO functional analysis offers the possibility to provide a GO functional classification and GO functional significance enrichment of DEGs. Using GO significant enrichment analysis, the major biological functions of DEGs, were determined [69,70].

KEGG (Kyoto Encyclopedia of Genes and Genomes) is a database for systematic analysis of genome information and gene function. The KEGG database allows the analysis of the metabolic pathways and functions of genes products, facilitating the investigation of genes and gene expression networks. KEGG incorporates data from genomes, chemical molecules, and biochemical systems, including metabolic pathways, drugs, diseases, and gene sequences. KEGG analysis was performed to evaluate the pathway significance enrichment and the hypergeometric test was used to find out that the pathway was significantly enriched in the DEGs compared with the whole genome background [71].

### 4.10. Key Modules and Hub Genes Identification

STRING (STRING. Available online: http://string-db.org 17 June 2019) was used to evaluate the hub genes by performing a gene network analysis. STRING contains a database able to predict and verify protein–protein interactions, including direct physical interactions and indirect functional relationships. It is based on the use of experimental data from content management literature and bioinformatic methods to predicts results. The biological methods employed for the analysis include chromosome proximity, gene fusion, phylogenetic tree, and gene co-expression of gene chip data. A scoring mechanism is used by the system to set the results obtained by different methods. STRING enables us to construct an interaction network combining the results of differential expression analysis with the interactions between the databases and the DEGs.

### 4.11. Statistical Analysis

Data are presented as mean ± SD of replicate. All the experiments were performed at least in triplicate. Data were analyzed using Student’s *t* test and the differences were considered significant for *p* < 0.05. Data analysis for flow cytometry was performed using FACSDiva software (BD-Bioscience Mountain View, CA, USA).

## Figures and Tables

**Table 1:** The top 15 hub genes enrichment with GO and KEGG.

Category	Term	Description	Gene Count	*p* Value
Biological Process (GO)	GO:0010629	Negative regulation of gene expression	12	2.33 × 10^−12^
Biological Process (GO)	GO:0090304	Nucleic acid metabolic process	12	5.05 × 10^−8^
Biological Process (GO)	GO:00427969	DNA demage response, detection of DNA demage	4	9.62 × 10^−8^
Biological Process (GO)	GO:0042276	Error-prone translesion synthesis	4	2.46 × 10^−6^
Biological Process (GO)	GO:0006297	Nucleotide-excision repair, DNA gap filling	3	3.52 × 10^−6^
Biological Process (GO)	GO:0070911	Global genome nucleotide-excision repair	3	4.76 × 10^−6^
Biological Process (GO)	GO:0000122	Negative regulation of transcription by RNA polymerase II	5	1.05 × 10^−3^
KEGG_PATHWAY	Hsa03010	Ribosome	11	2.96 × 10^−20^
